# EACLIPT Statement on the Importance of Science and Evidence-Based Treatment for Mental Health

**DOI:** 10.32872/cpe.18031

**Published:** 2025-05-28

**Authors:** Chantal Martin-Soelch, Claudi Bockting, Josefien Breedvelt, Lisbeth Frostholm, Nina Heinrichs, Colette Hirsch, Agnieszka Popiel, Winfried Rief

**Affiliations:** 1 Department of Psychology, University of Fribourg, Fribourg, Switzerland; 2 AmsterdamUMC, Department of Psychiatry, Amsterdam Public Health, University of Amsterdam, Amsterdam, The Netherlands; 3 The Centre for Urban Mental Health, University of Amsterdam, Amsterdam, The Netherlands; 4 Institute for Advanced Study, University of Amsterdam, Amsterdam, The Netherlands; 5 Department of Child and Adolescent Psychiatry, Institute of Psychiatry, Psychology and Neuroscience, King's College London, London, United Kingdom; 6 Department of Clinical Medicine, Aarhus University, Aarhus, Denmark; 7 Department of Functional Disorders, Aarhus University Hospital, Aarhus, Denmark; 8 Department of Psychology, Bielefeld University, Bielefeld, Germany; 9 Department of Psychology, King’s College London, London, United Kingdom; 10 Institute of Psychology, SWPS University, Warsaw, Poland; 11 Department of Clinical Psychology and Psychotherapy, University of Marburg, Marburg, Germany

The European Association for Clinical Psychology and psychological treatment (EACLIPT) expresses deep concern regarding current trends concerning science, evidence-based treatment, and the potential threats to mental health.

Recent developments have highlighted a troubling trend of undermining and censuring scientific research and evidence-based practices and propagating false information in medical and psychological care. This shift poses significant risks to the well-being of individuals who rely on scientifically validated treatments for their mental health conditions.

It is crucial to ensure that mental health care remains grounded in rigorous scientific evidence to provide the best possible outcomes for those in need.

The spread of false or misleading information can undermine public trust in legitimate medical research and treatments, leading individuals to seek unproven or harmful alternatives. This is especially concerning for mental disorders, where the complexity of conditions and treatments already makes it challenging to identify solid, evidence-based research. Disinformation can exacerbate these difficulties, creating confusion and skepticism around scientifically validated therapies. Consequently, patients may struggle to access effective care, and healthcare providers may face increased barriers in delivering accurate information and support. Combating disinformation requires robust efforts to promote media literacy, enhance transparency in research, and ensure that credible sources are easily accessible to the public.

We are also deeply concerned about the impact of the current geopolitical situation worldwide on the mental health of individuals. The heightened political tensions, divisive rhetoric, armed conflicts and climate changes have created an environment of uncertainty and stress, which can exacerbate mental health issues.

We call for a renewed commitment to science and the well-being of individuals affected by mental health issues. Together, we can ensure that mental health care remains effective, compassionate, and grounded in the best available evidence. EACLIPT stands in solidarity with mental health professionals and advocates who are working tirelessly to protect and promote evidence-based practices.

